# Beta-blocker exposure and dobutamine hyperaemia in heart failure with reduced ejection fraction

**DOI:** 10.1093/ehjimp/qyag167

**Published:** 2026-10-06

**Authors:** Philip Hasbak, Ronan M G Berg

**Affiliations:** Department of Clinical Physiology and Nuclear Medicine, Rigshospitalet, Copenhagen University Hospital, Blegdamsvej 9, Copenhagen DK-2100, Denmark; Department of Clinical Physiology and Nuclear Medicine, Rigshospitalet, Copenhagen University Hospital, Blegdamsvej 9, Copenhagen DK-2100, Denmark; Department of Clinical Medicine, Faculty of Health and Medical Sciences, University of Copenhagen, Copenhagen, Denmark; Centre for Physical Activity Research, Rigshospitalet, Copenhagen, Denmark

**Keywords:** myocardial blood flow, dobutamine stress, adenosine, heart failure, beta-blockade

Shergill et al. compared adenosine and dobutamine stress in patients with heart failure with reduced ejection fraction (HFrEF) and found that adenosine produced a greater coronary hyperaemic response, whereas dobutamine produced a larger peripheral haemodynamic response.^1^ While the authors discuss intrinsic beta-adrenergic desensitization in heart failure, the possible impact of beta-blocker exposure deserves attention.

In their study, 77.4% (41/53) of patients with HFrEF were prescribed a beta-blocker compared with 20% (2/10) of control subjects.^1^ Although participants were advised to withhold rate-limiting medication for 48 h, adherence was not strictly controlled. Because beta-blockade affects dobutamine signalling more directly than adenosine signalling, any residual beta-blockade may therefore have had a greater effect on the coronary hyperaemic response to dobutamine compared with adenosine. This may be particularly relevant in HFrEF, where beta-1 receptors are selectively down-regulated and beta-adrenergic responsiveness is reduced.^2^ Consistent with this, Colucci et al. found the maximal inotropic response to intracoronary dobutamine in severe heart failure to be only 37% of that in subjects without cardiac dysfunction.^3^

There is also the issue of beta-blocker withdrawal itself. Withdrawal may not be pharmacologically neutral: in healthy volunteers, beta-adrenergic receptor density in circulating lymphocytes rose after chronic propranolol administration,^4^ the surrogate finding underlying the receptor up-regulation thought to mediate withdrawal hypersensitivity. Atropine was allowed during dobutamine stress to achieve 85% of age-predicted maximal heart rate, but the proportion requiring atropine was not reported.^1^ Of note, beta-blockade reduces the chronotropic response to dobutamine and increases the need for atropine.^5^ Achieving the target heart rate after atropine therefore does not necessarily indicate an equivalent response to dobutamine.

A paired study design cannot account for a medication effect that differs between the two stressors. This is particularly relevant to the comparison with controls, where dobutamine MPR was 1.74 in HFrEF and 3.07 in controls.^1^ We would therefore be interested to know how many participants withheld beta-blockers, how often atropine was required, and whether the dobutamine response was similar among the 12 patients not prescribed beta-blockers. Given the size of that subgroup, adding beta-blocker prescription as a covariate to the segmental mixed-effects model would probably be more informative still. These data would help clarify whether the lower dobutamine response reflects HFrEF physiology alone or was partly influenced by beta-blocker exposure.

## Data Availability

No new data were generated or analysed in support of this research. All data discussed are taken from the published articles cited in the reference list.

## Lead author biography

Philip Hasbak is Chief Physician and Head of Nuclear Cardiology at the Department of Clinical Physiology, Nuclear Medicine and PET, Rigshospitalet, Copenhagen University Hospital, and clinical associate professor at the University of Copenhagen. His work centres on quantitative myocardial perfusion imaging with rubidium-82 PET, in particular myocardial blood flow and flow reserve in coronary microvascular disease, and on multi-tracer PET protocols in cardiac sarcoidosis and amyloidosis. He supervises several PhD projects in cardiac PET and has a longstanding interest in sex differences in coronary physiology.
